# Herbal Hepatotoxicity: Clinical Characteristics and Listing Compilation

**DOI:** 10.3390/ijms17050588

**Published:** 2016-04-27

**Authors:** Christian Frenzel, Rolf Teschke

**Affiliations:** 1 Department of Medicine I, University Medical Center Hamburg Eppendorf, Martinistrasse 52, D-20246 Hamburg, Germany; 2 Department of Internal Medicine II, Division of Gastroenterology and Hepatology, Klinikum Hanau, Teaching Hospital of the Medical Faculty, Goethe University Frankfurt/Main, 63450 Hanau, Germany; rolf.teschke@gmx.de

**Keywords:** hepatotoxicity, herbal drug, herb induced liver injury (HILI), herbal hepatotoxicity

## Abstract

Herb induced liver injury (HILI) and drug induced liver injury (DILI) share the common characteristic of chemical compounds as their causative agents, which were either produced by the plant or synthetic processes. Both, natural and synthetic chemicals are foreign products to the body and need metabolic degradation to be eliminated. During this process, hepatotoxic metabolites may be generated causing liver injury in susceptible patients. There is uncertainty, whether risk factors such as high lipophilicity or high daily and cumulative doses play a pathogenetic role for HILI, as these are under discussion for DILI. It is also often unclear, whether a HILI case has an idiosyncratic or an intrinsic background. Treatment with herbs of Western medicine or traditional Chinese medicine (TCM) rarely causes elevated liver tests (LT). However, HILI can develop to acute liver failure requiring liver transplantation in single cases. HILI is a diagnosis of exclusion, because clinical features of HILI are not specific as they are also found in many other liver diseases unrelated to herbal use. In strikingly increased liver tests signifying severe liver injury, herbal use has to be stopped. To establish HILI as the cause of liver damage, RUCAM (Roussel Uclaf Causality Assessment Method) is a useful tool. Diagnostic problems may emerge when alternative causes were not carefully excluded and the correct therapy is withheld. Future strategies should focus on RUCAM based causality assessment in suspected HILI cases and more regulatory efforts to provide all herbal medicines and herbal dietary supplements used as medicine with strict regulatory surveillance, considering them as herbal drugs and ascertaining an appropriate risk benefit balance.

## 1. Introduction

There are worldwide efforts aiming to develop drugs from natural products. Such successful drug developments were recently honored by awarding the Nobel Prize for Medicine and Physiology 2015 for the discovery of two natural products that brought breakthroughs in the therapy of tropical parasitic diseases affecting millions of individuals worldwide. Youyou Tu received the price for her discovery of artemisinin for the treatment of malaria, and William C. Campbell and Satoshi Ōmura for their discovery of the avermectins for the treatment of helminthic diseases. In the context of herbal medicine, artemisinin is derived from the herbal Traditional Chinese Medicine (TCM) *Artemisia annua*, known for more than two thousand years as a Chinese herbal medicine for various ailments [1,2,3]. Artemisinin and its derivatives such as artesunate and arthemeter are successfully used to treat patients with malaria alone or in combination with other antimalarial drugs, and they are also effective against cancer cells and schistosomiasis [3]. Many other drugs have been developed from herbs of traditional or modern medicine [4]. Among these drugs are acetylsalicylic acid derived from the willow tree, atropine from *Duboisia myoporoides*, belladonna from *Atropa belladonna*, casanthranol from *Rhamnus purhiana*, cholchicine from *Colchicum autumnale*, digitoxine from *Digitalis purpurea*, digoxine from *Digitalis lanata*, ephedrine from *Ephedra sinica*, etoposide from *Podophyllum peltatum*, morphine from *Papaver somniferum*, paclitaxel from *Taxus brevifolia* or *Taxus baccata*, papain from *Carica papaya*, papaverine from *Papaver somniferum*, quinine from *Cinchona* species, reserpine from *Rauwolfia serpentine*, and vincristine from *Catharanthus roseus*. In the future, herbal medicinal products will have a potential to advance drug discovery and development in a major world market, which stands at about $ 83 billion and Europe accounts for over 50% of the total [5]. Impressing data are reported for China, where the use of herbal medicines represents around 40% of all health care services [6]. In other countries such as Australia, Canada, USA, Belgium, and France, the percentage of the population that has used herbal medicines at least once is estimated at 38%–75%.

Key issues of presently available and newly developed herbal medicines will have to focus on questions as to whether the benefit risk balance is appropriate [7] and on monitoring safety [6]. This should be associated with mandatory causality assessment of adverse reactions [6,7,8], especially related to the liver as the target organ [8,9,10,11]. Clinical and causality assessment in patients with suspected herbal hepatotoxicity or its synonym herb induced liver injury (HILI) is facilitated as specific approaches are identical or at least very similar to those of drug induced liver injury (DILI) [8,12].

In this review article, we will discuss typical features of HILI cases and provide a list of published case reports on HILI by various herbs including herbal TCM. We will also consider the problem of valid causality assessments and propose clinical and regulatory strategies to minimize the risk for patient with assumed HILI.

## 2. Data Sources and Searches

### 2.1. Search Terms

We searched the PubMed database to identify publications of case reports, case series, and review articles for the following terms: “herbs”, “herb induced liver injury”, and “herbal hepatotoxicity”. This allowed the identification of publications on herbal hepatotoxicity and herb induced liver injury which each provided hits of around 280,000 and 3,010,000, respectively. Our search was then extended further using additional keywords denoting herbal modern medicine and herbal traditional Chinese medicine, TCM, providing additional hits. The first 100 hits of publications in each category were commonly considered.

In addition, to ensure that articles of high quality were considered, we used our actualized personal scientific archives, which contain original full-length publications relating to HILI covering the years from 1990 to early 2016.

### 2.2. Data Extraction

Prior to our analysis, the publications were assessed regarding their scientific quality. To meet requirements, articles had to include a clinical case description with history and symptoms, provide laboratory data and delineate the outcome of HILI. Publications of relevance and good quality were considered for evaluation. Our search focused on publications in English language, but few reports in other languages were also considered if they were of significant clinical importance and added to present knowledge. The literature search was limited since many reports of HILI by herbal TCM were in Chinese without an English abstract. Consequently, these Chinese publications were outside the focus of our present review article. Publications were also searched manually for additional publications not yet identified. The literature search ended on 15 February 2016.

## 3. Variability of Herbal Product Types

Among the most frequently used herbal products in the world are green and black tea [13], herbal traditional medicines [8,9,14], and herbal dietary supplements (HDS) [11], while the proportion of herbal drugs manufactured and controlled according to regulation guidelines appears to be smaller [6,14]. With the exception of the popular green and black tea that are commonly well tolerated if not consumed as tea extract [7], the use of the other herbal products may cause various rare adverse effects [6] including serious ones affecting the liver [8,9,10,11]. The regulatory status of these herbal products differs from country to country [6,11,14].

## 4. Herbal Product Quality

With the exemption of most herbal drugs under strict regulatory surveillance, some other herbal products that are manufactured for human use may suffer from quality problems (Table 1) [15]. Adherence to the quality criteria of current Good Agricultural Practices (cGAPs) and current Good Manufacturing Practices (cGMPs) is mandatory to ensure consumer safety. It is often speculative and unclear to what extent a problematic product quality is responsible for a liver disease of a patient who consumed such a product if HILI is not established as valid diagnosis.

### 4.1. Plant Identification and Its Parts

Any herbal product needs a clear definition and identification of plant family, subfamily, species, subspecies, and variety. This information should follow classical botanical descriptions and requirements for any herb used. Disregard may cause variation in plant family and species, contributing to the overall batch and product variability. Information of the individual herb identification items is best provided by the manufacturers in the package leaflet that should be available to the physician who suspects HILI. Such leaflets usually contain the name of the herbal product and the address of the manufacturer who will provide additional information upon request. Therefore, all essential data of herb identification and the herbal product should be available before reporting HILI case details as spontaneous reports to regulatory agencies or as case report publication. However, pitfalls are evident already at this stage of case evaluation [15].

### 4.2. Misidentifications, Adulterants, and Impurities

Plant misidentifications, adulterants, and impurities are known for a long time and still are key issues for herbal product quality [15,16,17,18,19,20,21,22]. Plant misidentification remains undisclosed for consumers who believe that the herbal product they use contains correct herbal ingredients as labeled. Herbal products containing misidentified plants impose a high risk for the unaware consumer. Chemical compounds as adulterants are problematic for the consumers if they are not labeled as such. Such unlabeled adulterants have to be classified better as unlabeled contaminants, implying a risky product. Impurities are mostly a consequence of disregarding cGAPs and cGMPs (Table 1).

#### 4.2.1. Misidentifications

A thorough regulatory report in Canada described analytical approaches in herbal products that were initially suspected as causative of some HILI cases, with the result that the suspected herb was not present in the products used by the affected patients [16]. This observation suggests that in a patient with suspected HILI causality cannot be ascertained without careful product analysis, which requires that the used herbal product is made available for testing. Mislabeling was also discovered in other herbal supplements after product analysis and comparing the results with the product labeling [22]. Green tea extracts (GTE) with catechins as their main ingredients have been implicated before as causes in some patients with HILI [23]. This led to regulatory statements by the United States Pharmacopeia [24] and analyses for catechins in herbal dietary supplements implicated in hepatotoxicity [22]. For 73 products, no GTE or catechins were identified on the label, implying that GTE was not considered as cause for the injury; yet for 29 (40%) of these products, catechins were detectable, which might have caused the liver injury [22]. Therefore, mislabeling may lead to erroneous causality attribution, unless product analysis clarifies its correct ingredients. The specific problems with herbal dietary supplements in the context of possible HILI has been well summarized: the myriad available and often multiple implicated products, batch-to-batch and product-to-product variability, and the potential for interactions among ingredients within a product or with other medications frequently confound attribution of injury to any one product or ingredient [22].

Herbal misidentification is a complex condition and a major clinical challenge as it may harm dramatically the health of consumers. Until 2008, overall, 41 cases from China with hepatic sinusoidal obstruction syndrome (HSOS), the former hepatic venoocclusive disease (HVOD), were reported and causality was attributed to the herbal TCM Jing Tian San Qi (*Sedum aizoon*, syn. Stonecrop), but causal attribution to *Sedum aizoon* was obviously incorrect. *Sedum aizoon* lacks unsaturated pyrrolizidine alkaloids (PAs), and when applied to experimental animals, HSOS did not emerge [25], strongly suggesting that for the reported HSOS cases, PAs are responsible, which are contained in and provided by another herb [26]. In line with this is another hepatotoxicity case from Hong Kong with HSOS that was also ascribed to *Sedum aizoon*, but it turned out to have been caused by the herbal TCM Shan Chi (*Gynura segetum*) [26]. The name and appearance of *Sedum aizoon* is similar to the one of *Gynura segetum*, but botanical differentiation was considered possible for experts [26]. Finally, studies comparing both herbs provided clear evidence for *Gynura segetum* as culprit for additional cases of HSOS as compared to *Sedum aizoon*. Respective studies in rats showed that *Gynura segetum* contains PA and may cause experimental HSOS as assessed by liver histology; in contrast, PAs were not found in *Sedum aizoon* [26]. In an earlier experimental study, a model of the HSOS was established by PAs derived from a herb described erroneously as *Sedum aizoon* [27], which again does not contain PAs [26,28,29]. This suggests that the described experimental model [27] was due to the action of a herb, which contains PAs, most likely *Gynura segetum* [26,28,29], rather than *Sedum aizoon* that lacks PAs [28]. Based on these well founded considerations, evidence for a hepatotoxic potential of Jing Tian San Qi is lacking. The herbal TCM *Sedum aizoon* should therefore no longer be listed as a hepatotoxic herb, as done until recently [30].

*Gynura segetum* was involved in other cases of herbal misidentification. In two Chinese women, HSOS emerged, which was induced by PAs of the herbal TCM *Gynura segetum* (syn. Ju Shan Qi, Ju Ye San Qi, San Qi Cao, Shan Chi) [31]. Additional six cases were suspected earlier [32,33]; in at least four of these, the culprit was the PA containing herb *Heliotropium lasiocarpum* rather than *Gynura segetum* [34].

All these examples illustrate the difficulties to incorporate the correct herb into the herbal product. Users may not consume what is labeled and *vice versa* may use products with unlabeled ingredients. Unquestionably, a more stringent regulatory surveillance will lead to herbal products of good quality, providing thereby an improved consumer safety.

#### 4.2.2. Adulterants and Unlabeled Contaminants

Heavy metals such as lead, mercury, cadmium, or arsenic may be found in some herbal medicines, added as adulterants due to the belief they could enhance the efficacy of the herbs in their products [19,21,35,36,37]. However, these heavy metals remain mostly unlabeled. In particular, lead is a regular constituent of traditional Indian remedies [35]. The incidence of contamination with heavy metals is unknown, but one study shows that 64% of samples collected in India contained significant amounts of lead, with corresponding figures for mercury (64%), arsenic (41%), and cadmium (9%) [35]. Consequently, cases of heavy metal poisoning in connection with Ayurvedic medicine use have been continuously reported [38]. However, there is also the note that despite being widely used, hepatotoxicity from only a few Ayurvedic medicine products has been reported in the literature [38], referring to four publications [39,40,41,42]. *Centella asiatica*, an Ayurvedic medicine used mainly for leprosy, has been reported to cause granulomatous hepatitis and cirrhosis [39,40]. Severe hepatitis from prolonged intake of herbal Indian Ayurvedic products used for vitiligo has been reported by our group [41]. Additionally, in the large randomized controlled trial of the Ayurvedic herbal combination product Liv.52 [42] that contains capers, wild chicory, arjuna, black nightshade, yarrow, and others for the treatment of alcoholic liver cirrhosis [38], no effect on survival was observed in Child class A/B patients, but liver-related mortality was significantly increased after evaluation of the two-year survival [38,42], suggesting a potential detrimental effect of the Ayurvedic product [38].

Hepatotoxicity in consumers of the herbal TCM mixtures Chaso and Onshido was ascribed to *N*-nitroso-fenfluramine, an unlabeled compound found in these slimming aid products that had been produced in China and sold in Japan and was discussed as an adulterant [43]. However, there is only little clinical or experimental evidence for a potential hepatotoxicity by this chemical compound, which was unlabeled and should better be classified as undisclosed contaminant [44,45,46], as discussed in detail in a previous report, which suggested that green tea extract as ingredient of these formulations is likely the causative agent [47]. Not provided by any supportive evidence is also the belief that 1.3-dimethylamine (DMAA), Aegeline, or both are hepatotoxic [11], which were labeled ingredients of other dietary supplements (DS) used by some liver patients, in whom overall hepatotoxicity causality for the DS themselves had to be denied [48,49,50].

Another group of adulterants refers to synthetic drugs, which are added to some herbal products to fortify therapeutic effects, but not all synthetic drug adulterants are labeled as such [15,17,18]. Adulterations with synthetic drugs was described especially for Chinese herbal medicines, with a long list of drugs commonly used for many indications in modern medicine [19,51]. Most disturbing was a study from Taiwan that showed that 24% of the samples were contaminated by at least one adulterant [16,51]. Overall, some of these adulterated drugs were harmful for the patients [51]. In this context, however, the question in these patients is as to whether the liver disease is a HILI by the consumed herbs or a DILI caused by the synthetic drug. As most synthetic drugs are well tolerated without liver injury by most individuals, DILI may be extremely rare in patients using these synthetic drugs contained in herbal products. Thus, herbs are more likely candidates for the toxic liver disease under these conditions.

It remains to be established to what extent also other problems of product quality such as plant misidentifications, adulterants and unlabeled contaminants are responsible for HILI, which must be clarified on a case by case basis. In fact, the possibility of contamination frequently confounds attribution of injury to any one product or ingredient [22].

#### 4.2.3. Impurities

Most nature based herbal products are confronted with some risks of impurities such as aflatoxinosis evolving during their agricultural and manufacturing process of TCM herbs [52,53] and non-TCM herbs such as kava, *Piper methysticum*, a South Pacific medicinal plant [54]. On theoretical grounds, aflatoxins may contaminate kava plants due to the humid conditions and high temperatures of the South Pacific region [54,55,56,57]. These and other issues of impurities as well as additional uncertainties about the kava product quality led to analyses, discussions [58,59], and a proposal for a kava quality standard [60] to ultimately ensure all quality requirements of kava as herbal anxiolytic medicine [61,62,63]. Subsequently, this goal was achieved by providing good quality of kava to be used in clinical trials to treat patients with anxiety disorders, and kava therapy was found to be effective and safe [64,65,66,67,68,69].

## 5. Pathogenetic Aspects of Herb Induced Liver Injury (HILI)

### 5.1. General Considerations

Specific events leading to HILI are mostly unknown, as HILI is primarily a human and not an animal disease; experimental models to study in detail the mechanisms leading to injury are therefore rarely available. For some TCM herbs known to cause liver injury, various toxic substances have been proposed and are listed as examples (Table 2) [70]. The pathogenesis of hepatotoxicity by TCM herbs containing unsaturated PAs has been elucidated in experimental studies, which showed hepatic microsomal cytochrome P450 (CYP) to be involved in the activation of PAs [71]. Similarly, and due to its experimental reproducibility in animals, the molecular pathogenesis of Germander (*Teucrium chamaedrys*) hepatotoxicity can easily be studied and transferred to human Germander hepatotoxicity [72]. Germander components are neoclerodane diterpenoids that are oxidized by the CYP 3A isoform into reactive metabolites. These deplete hepatic stores of glutathione and cytoskeleton associated protein thiols, form plasma membrane blebs, and cause apoptosis of liver cells [72,73]. Many other reports suggest chemical ingredients as causes for various HILI cases, but these suggestions are often speculative for human HILI.

As opposed to HILI, many more studies on pathogenetic aspects have been done in patients with DILI but it is uncertain whether such results are transferrable to HILI. For instance, recent DILI relevant data on actual pathogenetic hallmarks are directed to: (1) genome-wide association studies, which identified genetic predisposition as a relevant risk factor for liver injury [73,74]; (2) human leucocyte antigen (HLA) genotype as a strong risk factor for DILI, likely involving a drug-peptide adducts presented to T cells, although HLA alleles are only associated with some forms of DILI [73]; and (3) non-HLA genetic risk factors, which are thought to play a contributory role, especially those related to drug metabolism, detoxification, and disposition [73,74]. 

Involved genes may result in polymorphisms of bioactivation pathways via the CYP systems (Phase I reactions), conjugation reactions (Phase II), and excretion and transport (Phase III) [74]. For some drugs, even a dual role of HLA and drug metabolism genes is under consideration [73]. Other pathogenetic aspects related to DILI are still under discussion, in particular risk factors such as high lipophilicity and high daily doses of oral medications [75,76].

### 5.2. Idiosyncratic and Intrinsic HILI

**Figure 1:**
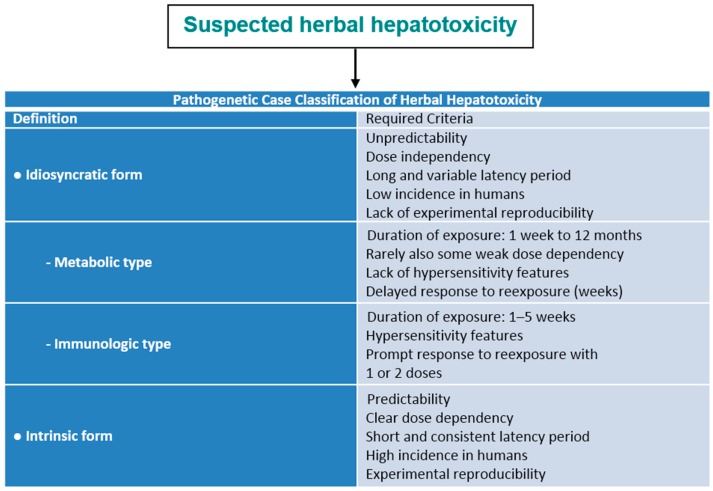
Pathogenetic classification of HILI. Data adapted from a previous report [8].

Every HILI case report should include data relevant for a pathogenetic case classification, using appropriate criteria that characterize the different types of HILI (Figure 1). In analogy to DILI, HILI is best described by its two types, the idiosyncratic HILI and the intrinsic HILI [77]. Idiosyncratic HILI is typically caused by herbs at therapeutic dosages through an idiosyncratic and thereby unpredictable reaction, which may be either metabolic or immunologic, as opposed to intrinsic HILI that develops from predictable reactions due to overdosed herbal products (Figure 1).

Because typical features show some overlap (Figure 1), few HILI cases may be ascribed to both idiosyncratic and intrinsic injury, especially when herbal product quality is different regarding toxic ingredients. Although valid data are lacking, it appears that most HILI cases are of the idiosyncratic rather than the intrinsic type. Overall, most plants are fairly well tolerated by humans, whether used as normal food, beverage, herbal drugs, or HDS.

#### 5.2.1. Idiosyncratic HILI Type

A good example of the idiosyncratic HILI type is the hepatotoxicity by Greater Celandine (GC) (Table 3) with its metabolic subtype [78] in reference to case details presented in previous reports [78,79]. GC hepatotoxicity is not reproducible in experimental animal models.

Another example is the hepatotoxicity by Indian Ayurvedic herbs (Table 4) [41]. This case represents again the idiosyncratic HILI type with its metabolic subtype. It is interesting to note that HILI by other Indian Ayurvedic herbs that are consumed not only in India but also worldwide are not commonly published in the English language, excepting perhaps herbs coantaining PAs.

Kava hepatotoxicity is primarily an idiosyncratic liver injury of the metabolic subtype in most cases, but it may also have features of an intrinsic injury type (Table 5) [80,81]. This dualism of typology is the result of various analyses [80,81,82,83,84,85,86,87,88,89,90,91,92,93]. In most kava products, toxic ingredients were likely absent, and their hepatotoxicity is based on idiosyncrasy in few susceptible individuals (Table 5). In few other kava products, toxic ingredients presumably are present, causing intrinsic hepatotoxicity in those few individuals who took these products [80,81,82,83,84,85,86,87,88,89,90,91,92,93]. The overall rarity of kava hepatotoxicity was also considered in a recent court trial and evaluated as a positive constellation of benefit and risk [93], opposing previous regulatory assumptions to the contrary [94].

#### 5.2.2. Intrinsic HILI Type

Some examples of typical intrinsic HILI are considered that are of major clinical or scientific interest. Among these are plants containing PAs such as *Crotalaria species* (Bush tea, Rattlebox), Gynura segetum, *Ilex paraguarensis* (Mate tea), *Symphytum* species (Comfrey), *Senecio* species (Groundsel), *Heliotropium* species, and *Compositae* species (Indian herbs) which cause HSOS as a specific form of liver damage [26,27,28,29,30,31,32,33,34,38]. Unsaturated PAs damage the sinusoid endothelial cells of the liver and reduce thereby the sinusoidal blood flow [38], which explains the typical clinical features of HSOS, caused for instance by *Gynura segetum* (Table 6) [28]. HSOS caused by unsaturated PAs is clearly dose dependent, thereby predictable, and hence preventable. Consequently, every consumer of these herbs containing PAs is at a dose dependent risk of HSOS. Plants containing PAs are among the most abundant poisonous plants affecting not only humans but also livestock and wildlife, with more than 6000 plant species containing PAs and about 3% of the world’s flowering plants containing PAs [95]. Human embryotoxicity by PAs resulting in fetal HSOS has been described in a newborn whose mother drank one cup of a tea containing PAs per day throughout pregnancy [95,96].

Other intrinsic HILI are caused by plants such as Germander [72], *Radix bupleuri* [97,98], *Polygonum multiflorum* [99], and green tea extracts (GTE) [13,22,23,24,100]. Germander hepatotoxicity is dose dependent, well described in detail, and is reproducible in mice [72]. Herbal TCM products containing more than 19 g dose of *Radix bupleuri* may increase the hepatotoxicity risk [97]; this dose dependency was confirmed in experimental animals and provided insights into some pathogenetic processes [98]. Hepatotoxicity by *Polygonum multiflorum* is also a classic example of intrinsic HILI [99]. Finally, GTE also shows a clear dose dependency, classifying thereby GTE hepatotoxicity as intrinsic HILI [13,22,23,24,100].

## 6. Clinical Features of HILI

PCP and clinicians are faced with the problem to identify patients with suspected HILI early in the evolving disease. However, clinical symptoms of patients with hepatotoxicity by herbal traditional and modern medicine are variable, mostly unspecific, and usually difficult to attribute to the liver, which delays early recognition of the unfolding liver injury [15,70]. In the context of clinical signs, two groups have to be differentiated, one group with HSOS by HILI from PA containing herbs and the other group with HILI unrelated to the use of PA containing herbs.

Patients with HSOS present with abdominal distension and pain, malaise and body weight gain due to hepatomegaly and ascites [29]. On a quantitative basis, typical clinical signs are shown as example for *Gynura segetum* causing HSOS (Table 6). The leading symptom of HSOS is ascites, which is otherwise rarely found in patients with HILI unrelated to PA use, except perhaps in end stage conditions. Ascites as the leading clinical sign must alert the physician considering HSOS as a diagnostic option. Discrediting this key finding has led in the past to abundant misdiagnoses and further harm to the patients, as detailed and discussed in many reports [25,26,27,28,29,30,31,32,33,34].

Other clinical signs prevail in patients with HILI unrelated to the use of PA containing herbs (Table 3, Table 4, Table 5) because ascites is uncommon and not reported in HILI cases by GC [78], Indian Ayurvedic herbs [41], and kava [80,81] as well as *Polygonum multiflorum* [99], or other TCM herbs [70,101]. In detail, patients with herbal TCM hepatotoxicity experience fatigue (67.3%), jaundice (60.3%), anorexia (58.0%), nausea (35.9%), and fever (19.3%), but signs such as rash, pruritus, and pale colored stools have also been reported [70]. In another study with established HILI by GC, symptoms were present in 15 of 16 reported cases [78]. Single or multiple symptoms were jaundice (*n* = 15), nausea (*n* = 6), fatigue (*n* = 5) anorexia (*n* = 3), dark urine (*n* = 3), pruritus (*n* = 3) ,vomiting (*n* = 2), dyspepsia (*n* = 1), bloating (*n* = 1), abdominal discomfort (*n* = 1), right upper quadrant pain (*n* = 1), epigastric pain (*n* = 1), unspecified abdominal pain (*n* = 1), pale stools (*n* = 1) and fever (*n* = 1) [78]. Patients with HILI may be asymptomatic with increased values observed by chance, mono-symptomatic, or poly-symptomatic, while jaundice is the best initially recognized symptom by the patient, facilitating the search for advice by the PCP [78]. In HILI cases unrelated to PAs, the chronology of symptoms commonly follows a particular stepwise pattern, as described for HILI caused by Indian Ayurvedic herbs, through an excellent observation by a patient (Table 4) [41]. Her symptoms started with pruritus, followed by loss of appetite, fatigue, nausea, vomiting, dark urine, light colored stools, until finally jaundice was recognized by her family physician; this sequence of symptoms stretched over almost four months under continued medication.

Latency periods defined as interval between start of herbal use and emerging symptoms or increased liver values are variable. Liver injury by herbal TCM develops slowly with clinical symptoms appearing between one week and one month [70], or up to 150 days [101]; with a longer latency period of four to 260 weeks for GTE [13,23], of one week to 24 months for other herbs such as kava [80,81], of 28 to 134 days for GC [78,79], or of seven months for Indian Ayurveda herbs [41].

## 7. Progress in Developing Valid Diagnostic Biomarkers

Numerous serologic markers exist, that enable a firm diagnosis of most liver diseases unrelated to HILI and DILI, for instance by assessing specific antibodies of viral hepatitis. New encouraging steps with the development of specific biomarkers for HILI are discussed [71] in reference to a sensitive and specific assay enabling the detection of reactive pyrrole-protein adducts in the serum of patients with HSOS. This disease was attributed to the Tusanqi preparation made erroneously with *Gynura segetum* containing PAs instead with *Segetum aizoon* lacking PAs [26]. The results of this assay show that the patient actually consumed a herb containing PAs, which are metabolized in the liver to a reactive PA metabolite, reacting with a protein and forming an adduct [71]. However, this assay does not prove that PAs have caused the hepatotoxicity in this particular patient, as supportive evidence is required in the clinical context. Measuring herbal toxins or their metabolites in the serum is useful in HILI cases in a setting of intoxication, if high levels of the herbal toxin are expected in the serum due to large amounts of the consumed herb, high cumulative doses, or a prolonged degradation of the toxic herbal chemical. These conditions apply to HILI cases of the intrinsic form but not to those of the idiosyncratic form, which accounts for most HILI cases. For idiosyncratic HILI, similar restrictions apply regarding circulating micro-RNA (mRNA), presently investigated in intrinsic DILI and detectable in fluids including the serum [102]. Omics technologies, including genomics, proteomics, and metabolomics might well change but not revolutionize our understanding in the diagnosis of intrinsic hepatotoxicity [103].

For idiosyncratic DILI, numerous genetic and nongenetic risk factors have been described as possible biomarkers to predict DILI in susceptible individuals [104], but whether these are useful to identify a person at risk for idiosyncratic HILI is unknown.

## 8. HILI Case Criteria

A case of acute HILI is defined by increased serum levels of ALT (alanine aminotransferase) of at least 5N and/or of ALP (alkaline phosphatase) of at least 2N, whereby N represents the upper limit of normal [12]. Both tests should be best performed simultaneously on the day of first presentation or suspicion. These thresholds were chosen to increase the specificity of causality assessment for hepatotoxicity, eliminate false positive cases, and substantiate hepatotoxicity causality with a high level of probability. Special care is needed, if ALT is within the normal range and ALP is increased, this should then be paralleled by increased γ-glutamyltranspeptidase or better 5′-nucleosidase to rule out isolated increase of ALP activity due to bone or another origin than hepatobiliary disease [12].

## 9. Liver Injury *versus* Adaptation

Hepatotoxicity criteria are to be applied for each case of suspected HILI [12]. Concern emerges whenever hepatotoxicity is suspected even if LTs were only marginally increased, not reported, or not available. These problems are common for cases of suspected HILI, presented for instance by the United States Pharmacopeia [105] relating to both black cohosh (BC) [106,107,108,109,110] and GTE [24,111], by the World Health Organization relating to kava [88,112], by the German regulatory agency BfArM (Bundesinstitut für Arzneimittel und Medizinprodukte) [94,113] relating to kava [81,114], or by the Drug Commission of the German Medical Association [115] relating to *Pelargonium sidoides* (PS) [116,117,118]. In contrast, published case reports receive the benefit of appropriate peer reviews, and thus the presented HILI cases provide relevant data, commonly reporting high values of aminotransferases and/or ALP and basic data support of potential hepatotoxicity, as shown also for some cases with a positive reexposure test [119]. In other HILI case series, however, criteria were documented incompletely; neglecting these aspects in effect invalidates the causality assessment.

Contrasting to severe liver injury with its high LTs [47], use of herbal TCM may also cause only mild LT increases [120] that remain below the threshold values of liver injury [12]. These conditions are considered as liver adaptation or tolerance, especially if herbal use is continued and LTs remain stable or return to normal range, but transition to severe liver injury is possible. Liver adaptation is commonly observed under a therapy with synthetic drugs such as statins and antituberculous medications, especially isoniazid, and is likely the consequence of metabolic events during enzymatic drug degradation [121]. Most herbal products are well tolerated by the majority of consumers [6]. Even under comedication with synthetic drugs, only slight increases of LTs are rarely observed in patients under treatment with herbal TCM; in some of these patients, pre-existing LT abnormalities were known but were obviously not considered as risk factors of HILI by herbal TCM [120].

## 10. HILI Case Classification

For cases of suspected HILI, hepatotoxicity case classification is mandatory to facilitate further evaluation of reexposure results and RUCAM assessments [12]. The classification of the liver injury pattern (also called phenotype) is essential and must be provided. Three different types of liver injury are to be considered: hepatocellular, cholestatic, and mixed. These types can readily be identified by initial measurement of ALT and ALP without the need of a liver biopsy result [12]. The ratio *R* is the basis for the classification and is calculated as the ALT: ALP, both activities measured at the time when liver injury is suspected and expressed as multiples of N, the upper limit of the normal range. Liver injury is hepatocellular if *R* ≥ 5; liver injury is cholestatic if *R* ≤ 2; and liver injury is mixed if 2 < *R* < 5. This classification of liver injury pattern clearly assigns each HILI case to RUCAM (Roussel Uclaf Causality Assessment Method), either for the hepatocellular injury, the cholestatic, or the mixed liver injury.

In a HILI case series of herbal TCM consisting of 27 patients, the pattern of liver injury was hepatocellular in 82% of the cases, cholestatic in 11%, and mixed in 7% [101]. In another small case series of HILI by herbal TCM, all 12 patients who experienced an unintentional reexposure had a hepatocellular type of injury [47].

## 11. RUCAM as the Most Used Method to Assess Causality

Causality for herbs in patients with suspected HILI is best achieved with RUCAM in its current version updated in 2016, which provides two scales, one for the hepatocellular type of injury and the other one for the cholestatic or mixed type of liver injury [12]. RUCAM is a well established tool to quantitatively assess causality in HILI and DILI cases (Table 7) and has many advantages compared to other causality tools, as discussed in detail in reference to other approaches that cannot substitute RUCAM [12]. Representing a structured, standardized, and validated diagnostic approach specific for hepatotoxicity, RUCAM attributes scores to individual key items that reflect the natural course of HILI. The scores provide a final quantitative grading of causality for each suspect herb in a case report (range of final scores from 14 to −3) of highly probable, probable, possible, unlikely, or excluded. This meets the requirements of clinicians in care for their patients with suspected HILI, to establish the diagnosis in time with a high degree of confidence. In many countries and for more than two decades, physicians, international registries, regulatory agencies, and pharmaceutical companies successfully applied RUCAM for suspected HILI, and the score is also used in case reports with increasing tendency [12].

RUCAM covers a broad spectrum of alternative diagnoses that must be excluded before HILI can be assumed as cause of liver damage: these include infections by various hepatotropic viruses, not limited to hepatitis A, hepatitis B, hepatitis C and hepatitis E virus (HEV) with specific parameters [12]. Equally important for RUCAM are infections by cytomegalovirus, Ebstein Barr virus, herpes simplex virus and varizella zoster virus, to be assessed by parameters such as PCR and IgM/IgG antibody titers also upon repetitive testing. RUCAM also pays special attention to HEV, a neglected infection in assessments of liver injury cases [48,49,50,122,123,124,125]. Even in a clinical trial, an infection by HEV may occur and create confusion, mimicking a DILI [126]. An additional problem exists in the United States, where the HEV issue remains disputed due to lack of FDA-approved HEV antibody tests that provide valid results [49,50,123,124]. These viral infections being the causes of pre-existing liver diseases need to be excluded in the diagnostic approach of HILI.

Overall, assessment with RUCAM provides transparency by presenting each RUCAM item with the appropriate score, which should be included in published cases to facilitate reassessment and discussions among experts [12]. RUCAM is user-friendly as it highlights possible differential diagnoses, so that the work-up of an individual patient can be achieved in the absence of a hepatology specialist.

An internationally harmonized, uniform approach of causality assessment is preferred, including a case narrative, RUCAM, and a checklist for differential diagnoses of HILI [12]; optionally, an expert panel may assist, reassessing the clinical case characteristics and the quantified RUCAM items obtained by the caring physician. This structured approach would help to ensure completeness und transparency of case data. It is also a chance for an internationally harmonized approach of causality assessment and improves the acceptance of published case reports or case series on HILI. It would be helpful if uniformity of HILI criteria including specific scoring was established worldwide so published data across countries, and their registries could be harmonized and easily interpreted across populations. Therefore, RUCAM should best be considered as a standardized approach for causality assessment of HILI cases, both for the attending physician and all later stages by experts if really needed [12]. Use of a single assessment method allows valid and reproducible comparisons of different assessment outcomes.

RUCAM also provides clear criteria for a positive unintentional reexposure [12]. Such positive result is considered a gold standard to establish the DILI diagnosis; this was also used to validate RUCAM as reliable diagnostic tool for causality assessment [127]. None of the other causality assessing methods such as the DILIN method, the WHO method, or the Naranjo scale used any gold standard for validation, and the two latter assessing approaches are not hepatotoxicity specific and hence obsolete [12].

## 12. Reexposure

Reexposure can only be involuntary since intentional reexposure is considered unethical due to the high risk of severe liver injury [12]. Although unintentional reexposure is rarely described in HILI cases, reported results are of utmost importance for individual case analysis, provided their results are positive and meet the required criteria (Table 8). 

In recent analyses of cases with HILI by herbal TCM, cases were compiled which underwent assessment by RUCAM, reexposure test, or both [128,129]. Positive reexposure test results ensured causality for only a few TCM herbs, for which HILI was described. For more herbs, a probable or highly probable causality grading by RUCAM was achieved and provided a clear causality of HILI. Regarding the reexposure tests, positive results are claimed in many case reports for individual TCM herbs, but criteria were not fulfilled, resulting in uninterpretable or even negative results [128,129]. For future cases, details of the essential criteria for positive test results should be provided to allow reevaluation.

## 13. Herbs with Established Causality for HILI

Some TCM herbs of this category are listed (Table 9), this also included green tea (La Chu) as its GTE [13,23,100]. For non-TCM herbs with established causality by RUCAM, positive reexposure test, or both, analyses were provided for GC (Table 3), kava (Table 5), Indian Ayurvedic herbs [41], Aloe, Chaparral, Germander, Mistletoe, Senna, and Skullcap [129]. Certainly many more herbal products will have to be added, awaiting further analyses. For this category but without providing criteria, proposals have been also made recently for Chaparral, Comfrey, Senna, and wild germander, in addition to the TCM He Shou Wu [9].

## 14. Questionable and Lacking Causality

The herbal TCM Ba Jiao Lian (*Dysosma pleianthum*) cannot be considered as hepatotoxic herb because essential diagnostic hepatotoxicity criteria were lacking in patients who used this TCM herb [47,130]. In detail, after herbal use at recommended doses, the patients manifested abnormal liver function tests associated with nausea, vomiting, diarrhea, abdominal pain, thrombocytopenia, leucopenia, sensory ataxia, altered consciousness and persistent peripheral tingling or numbness. However, the increase of the aminotransferases was marginal, with preference of AST rather than ALT. The AST increase could reflect isolated damage of the mitochondria around the hepatic central vein or muscular damage, because of the associated increase of creatine phosphokinase, findings not in support for a clinically relevant toxic liver disease [47]. Clear evidence against a hepatotoxic potential was also provided for Jing Tian San Qi (*Sedum aizoon*) as another herbal TCM [47], based on the results of recent studies showing that in patients with HSOS, the hepatotoxic PAs in the herbal TCM Shan Chi (*Gynura segetum*) were responsible rather than the misidentified *Sedum aizoon* lacking these alkaloids [25,26,27,28,29,31]. RUCAM based causality was lacking for *Pelargonium sidoides* [116,117,118], OxyELITE Pro [48,49,50], and BC [106,107,108,109,110], for which a lack of hepatotoxicity was confirmed by meta-analysis of randomized controlled clinical trials [131].

## 15. Alternative Diagnoses

Analyses have shown that liver damage in suspected HILI can often be explained by alternative causes [48,49,50,132], a problem also reported for DILI cases [133]. The problem of missed diagnoses is multifaceted and caused by incomplete case data collection, poor case data analysis, incomplete transfer of case data from medical files to the manuscript, and unjustified upgrading of causality scores [48,49,50,132]. Clinical problems can emerge, when specific effective treatment and preventive measures that would have been available for some patients with alternative diagnoses are withheld [48,49,50]. The issue of alternative causes was assessed in 23 publications that comprised 573 cases of initially suspected HILI [132]. In this analysis, alternative diagnoses were evident in 278 cases (48.5%) misdiagnosed as HILI. Among the missed diagnoses were hepatitis by various viruses (9.7%), autoimmune liver diseases (10.4%), nonalcoholic and alcoholic fatty liver disease (5.4%), liver injury by comedication (DILI or other HILI) (43.9%), and liver involvement in infectious diseases (4.7%). Biliary and pancreatic diseases were frequent alternative diagnoses (11.5%). Pre-existing liver diseases including cirrhosis (9.7%) were additional confounders. Other diagnoses were rare, but possibly relevant for the individual patient [132]. These results are alarming, because HILI is by mainstream definition a diagnosis of exclusion, and it does not appear that this aspect was appropriately considered by some physicians. The use of RUCAM will certainly reduce the risk of missed diagnosis in future cases of suspected HILI [12].

As a reminder for the clinician that many diagnoses exist as alternatives to HILI, rare causes of liver disease are included in a checklist of differential diagnoses [12] These other diagnoses have to be considered, excluded or verified in the context of clinical data and importance, financial resources, and benefit for the patient. Establishing alternative causes contributes to the accuracy of RUCAM and often provides clues to possible specific therapies.

## 16. Confounding Variables

In the past, many case reports about HILI presented incomplete case data or neglected causal relation between suspected herbal compound and liver damage. As expected under these conditions, major shortcomings prevail that are to be considered as confounding variables impeding a valid causality in perhaps most of the reported cases (Table 10) [14,16,22,25,26,27,28,29,48,49,50,99,105,106,107,134,135]. For instance, chances were missed by a recent review article about HILI by *Polygonum multiforme* describing excellent details of the used herbal products, daily doses, and treatment duration among others; causality for the herb was not assessed and it remained unclear whether alternative causes were excluded (Table 10) [99]. Confounding variables may result in false high signal cases and case overreporting by overdiagnosing.

## 17. Listing Compilation of Published Reports with Potentially Hepatotoxic Herbs

Numerous case reports and case series have been published, suggesting that TCM herbs may have caused liver injury but results of a valid causality assessment were rarely provided. The presented listing compilation is therefore tentative and awaits further analyses including valid causality assessments such as by RUCAM for most of the published case reports and case serries (Table 11) [22,23,24,40,99,100,136,137,138,139,140,141,142,143,144,145,146,147,148,149,150,151,152,153,154,155,156,157,158,159,160,161,162,163,164,165,166,167,168,169,170,171,172,173,174,175,176,177,178,179,180,181,182,183,184,185,186,187,188,189,190,191,192,193,194,195,196,197,198,199,200,201]. Additional information and references for each herb under consideration is provided in two recent reports [8,128]. Another listing compilation is provided for non TCM herbs with reported and suspected liver injury (Table 12) [22,23,24,32,40,41,72,100,111,136,137,149,158,171,172,173,174,175,176,177,178,180,189,202,203,204,205,206,207,208,209,210,211,212,213,214,215,216,217,218,219,220,221,222,223,224,225,226,227,228,229,230,231,232,233,234,235,236,237,238,239,240,241,242,243,244,245,246,247,248,249,250,251,252,253,254,255,256,257,258,259,260,261,262,263,264,265,266,267,268,269,270,271,272,273,274,275,276,277,278,279,280,281,282,283,284,285,286,287,288,289,290,291,292,293,294,295,296,297,298,299,300,301,302,303,304,305,306,307,308,309, 310, 311], which is adapted from a previous publication that provides additional data and references for each herb [8].

## 18. HILI Outcome and Management

### 18.1. Natural Course and Discontinuation of Herbal Use

As expected, little firm information exists of the natural course of HILI under continued herbal use despite evident symptoms, with the exception of perhaps one single study reporting details of a HILI case series [78]. In this analysis, ignorance of symptoms with corresponding delay of therapy discontinuation was observed in patients with severe HILI by GC. This study cohort consisted of 16 patients with established RUCAM-based causality gradings of “probable” or “highly probable”. In 15 of these patients (93.8%), jaundice was the leading symptom [78], in line with a severe clinical course according to the criteria of Hy’s law. Among the GC cohort patients, the accurate latency period to first symptoms was assessable in 13 patients; in eight of these, the latency period was identical with the duration of GC intake signifying that GC use was stopped after the appearance of first symptoms. In the remaining five cases with overt symptoms, treatment with GC was continued over a period of up to seven months but outcome was favorable, contrary to clinical expectations [78]. Such a good prognosis was not observed in a DILI case series in which the delayed cessation of the antituberculous drug was associated with a high risk of mortality [312]. Among 13 patients with continued isoniazid use for more than seven days, seven patients required a liver transplantation or died. Despite these differences of outcome depending on the offending product, intake of the herbal product must be stopped in cases of suspected HILI as a precautionary measure, especially since the efficacy of most herbal treatments has not validly been established by evidence based studies [7]. HILI commonly improves spontaneously upon cessation of the offending herb.

### 18.2. Severe HILI and Hy’s Law

Severe clinical courses of HILI with acute liver failure (ALF) are extremely rare but may require orthotopic liver transplantation to circumvent a lethal outcome; the underlying risk factors are largely unknown [144]. Preventive measures are not available in these HILI cases that are caused likely by an idiosyncratic reaction in the majority of patients (Figure 1). Jaundice is one of the cardinal symptoms of severe HILI and likely associated with a high risk of lethality, at least in DILI [313] cases but not yet firmly determined in HILI. For quantitative risk management of HILI, helpful recommendations may be derived from Hy’s law of the late Hyman Zimmerman, based on a 1 in 10 mortality risk of DILI if the following three criteria in short are met in hepatocellular liver injury: (1) serum ALT or AST >3N; (2) serum total bilirubin elevated to >2N; and (3) no other reason can be found for the combination of increased aminotransferases and bilirubin [313]. Patients with signs of impending acute liver failure (e.g., coagulopathy and encephalopathy) should be transferred to a specialized hepatology unit. Up to now, prognostic markers to predict the outcome and indicate, that a patient may require liver transplantation, are not evaluated in HILI.

### 18.3. Adaptation

Liver adaptation under herbal use requires LT surveillance [120]. This is mandatory due to the potential transition to severe injury. To be cautious, cessation of herbal use is recommended for the safety of the patients on a case by case basis, especially if indication is unclear and treatment was ineffective so far.

## 19. HILI Outbreaks

Several publications report HILI outbreaks related to consumption of food contaminated by plants, which contain unsaturated PAs [314,315,316,317]. For example, following a two-year period of severe drought, a very large number of patients with massive ascites and emaciation were observed in northwestern Afghanistan [314]. Clinico-pathological studies showed that these were typical cases of HSOS. The outbreak was caused by consumption of bread made from wheat contaminated with seeds of *Heliotropium* plants, which were shown to contain PAs. Examination of 7200 inhabitants from the affected villages showed evidence of liver disease in 22.8%. Clinical improvement was observed in thirteen cases after three to nine months, and in three cases liver biopsies showed almost complete disappearance of initial abnormalities [314]. A more recent outbreak of HSOS was reported from Western Afghanistan, associated with exposure to wheat flour contaminated with PAs, but the incriminated plant remained unclear [315].

Two other outbreaks of HSOS were reported from India [316,317]. One of these was probably caused by consumption of cereals mixed with seeds of a plant (*Crotalaria* sp.) containing PAs, which occurred in the Sarguja district of India end of 1976; among the 67 recorded cases, 42% of the patients died [317].

## 20. Regulatory Issues

Consumers are confronted with abundant herbal products, encompassing herbal drugs and herbal dietary supplements, not all of which are under tight control by government regulatory agencies. Most of these products are likely safe, others are certainly not. Based on careful randomized controlled trials, a favorable benefit-risk profile has been shown only for a limited number of these products, including Artemisia annua with its ingredient artemisinin used to efficiently treat patients with malaria. Interestingly, alone in the United States, more than 50,000 dietary supplements were marketed between 1995 and 2015 [11]. This calls for some product restriction. Preference should be given to herbal products that are to be used as herbal medicines or herbal drugs and fulfill global regulatory requirements of efficacy and safety.

## 21. Conclusions

Herbal use is highly appreciated around the world to treat various health conditions. Many herbal products are easily accessible via the internet and thus can escape regulatory surveillance. Concomitantly, herb induced liver injury emerges as a clinical problem and can evolve into acute liver failure in rare cases. Clinical signs are often non-specific and there is no diagnostic feature that delineates HILI from alternative causes of liver damage. Therefore, the diagnosis of HILI is one of exclusion and the RUCAM score can aid the clinician to establish causality. Furthermore, assessment according to RUCAM facilitates evaluation of published cases and provides useful information for authorities to regulate the marketing of herbal products. Clinical studies to evaluate the benefit/risk balance of herbal drugs are encouraged. Finally, all herbal products and herbal dietary supplements used as medicine should be under a more strict regulatory surveillance, considering these products as herbal drugs.

## Figures and Tables

**Table 1:** Minimum quality requirements for herbs, herbal drugs, and herbal dietary supplements in the context with suspected herb induced liver injury (HILI) cases.

Item	Product Quality Specifications
Herbal product declarations	Declaration of the manufacturer with address, phone and fax number, e-mail Expiration date of the herbal drug and herbal supplement Batch number Correct labelling of all ingredients
Herbal product definitions	Definition of plant family, subfamily, species, subspecies, and variety Definition of plant part Definition of used solvents and solubilizers
Herbal quality standards	Exclusion of impurities, adulterants, and misidentifications Minimum or lack of batch to batch variability Minimum or lack of product to product variability Lack of variety to variety variability Current Good Agricultural Practices (cGAPs) Current Good Manufacturing Practices (cGMPs) Regulatory surveillance
Consumer information	Detailed recommendation for indication and contraindication Advice for daily dose and maximum use duration

Adapted from a previous report [8]

**Table 2:** Some examples of suspected toxic compounds as suggested causes of hepatotoxicity by herbal Traditional Chinese Medicine (TCM).

Chinese Name	Scientific Name	Tentative Hepatotoxic Components
Ai Ye	*Artemisia argyi*	Volatile oil
Bi Ma Zi	*Rhicinus communis*	Ricin, toxic proteins
Cang Shan	*Xanthium*	Glycosides (kaurene), diterpenoids
Chang Shan	*Dichor febrifuga Lour*	Alkaloids (dichroine)
He Huan Pi	*Albizia julibrissin*	Glycosides (saponine)
He Shou Wu	*Polygonum multiflorum*	Anthraquinones
Huang Yao Zi	*Discorea bulbifera L*	Glycosides (steroids, diosgenin), diterpenoids-lactones
Ku Lian Zi	*Melia azedarach*	Glycosides (tetranortriterpenoids)
Lei Gong Teng	*Tripterygium wilfordii hook F*	Glycosides (tripterygium), diterpenoid-lactones
Qian Li Guang	Senecio scandens	Pyrrolizidine alkaloids
Shan Lu	*Phytolacca acinosa Roxb.*	Alkaloids (phytolaccine)
Xiang Si Zi	*Abrus Precatorius*	Abrin

Data are derived from a published report by Ma *et al.* [70].

**Table 3:** Clinical characteristics of HILI by Greater Celandine.

Items	Clinical Characteristics of HILI by Greater Celandine (GC)
● Background	1. Clinical characterization of HILI by GC as a specific disease entity was feasible and based on 16 patients with liver disease and high causality levels for GC;
● RUCAM	2. RUCAM based causality for GC was graded highly probable in 4 patients and probable in 12 patients;
● Comedication causality assessment by RUCAM	3. Among these 16 patients, there was an additional RUCAM based causality for comedication with curcuma graded as possible, for comedication with *Lycopodium serratum* graded as probable, and for biliary disease graded as possible;
● Positive reexposure result	4. The existence of GC HILI has been verified by a positive reexposure test in two patients;
● Age and gender	5. Ages of the 16 patients ranged from 32 to 69 years with an average of 54.7 years, and the ratio of females: males was 10: 6;
● High comedication rate	6. Comedication with synthetic or herbal drugs and dietary supplements and herbal mixtures was used in the majority of assessable cases;
● Chelidonine dose adherence	7. On average, the patients used 10 mg chelidonine daily, with no reported overdose in any of the cases;
● Variable treatment duration	8. Treatment duration was 3 weeks to 9 months with an average of 2.4 months;
● Continued GC use despite symptoms	9. Latency period until first symptoms was 3 weeks to 4.5 months with an average of 1.7 months, which was considerably shorter than the treatment length;
● Jaundice as major symptom of GC induced HILI	10. Jaundice was the most frequently reported symptom, rarely also weakness, anorexia, nausea, vomiting, abdominal pains, dark urine, pale stools, and itching;
● Hepatocellular liver injury	11. High serum activities are found for ALT but not for ALP, suggestive of hepatocellular liver injury in patients with GC HILI;
● Liver histology	12. Histology showed predominantly liver cell necrosis and hepatitis;
● Favorable outcome	13. Outcome was favorable in all 16 patients, with lack of both acute liver failure and requirement of a liver transplant;
● Good prognosis despite continued GC intake	14. In one patient, good prognosis was sustained even after 7 months of continued GC use despite presence of emerging GC HILI;
● Idiosyncratic liver injury with its metabolic subgroup	15. GC HILI usually represents the hepatocellular and idiosyncratic type of liver injury with its metabolic subgroup, characterized as acute clinical course;
● Individual culprits still undetermined	16. The underlying mechanism(s) leading to GC hepatotoxicity as well as possible culprit(s) are still unknown;
● Safety concern	17. In cases of liver disease, causality for GC was verified and creates concern regarding safety of patients;
● GC HILI likely with low incidence	18. Lacking valid epidemiologic data, but incidence of GC HILI is likely low.

The data are based on cases of 16 patients with GC hepatotoxicity with highly probable or probable causality levels reported in a publication [78]. Abbreviations: ALT, Alanine aminotransferase; AST, Aspartate aminotransferase; GC, Greater Celandine; HILI, Herb induced liver injury; PAs, Pyrrolizidine alkaloids; RUCAM, Roussel Uclaf Causality Assessment Method.

**Table 4:** Typical follow-up of symptoms in a case of HILI by Indian Ayurvedic herbs.

Conditions	Symptoms
● Herbal use for overall 9 months, with full daily dose as recommended for the initial 7 months.	Well-being during first 6 months of treatment with Indian Ayurvedic herbs for her vitiligo. Pruritus as first symptom after herbal treatment for 6 months, with subsequent symptoms step by step: Loss of appetite, fatigue, nausea, and vomiting several times per week.
● After herbal use for 7 months, dose reduction to 50% for the remaining 2 months of treatment. Dose reduction was the patient’s decision. The clinic in India, which distributed this herbal medicine via internet, denied an association with the described symptoms.	Dark urine after use of herbs for 7 months. Reduced daily dose led to disappearance of pruritus but other symptoms persisted. Light-colored stool as new symptom appearing 8 months after initiation of the herbal treatment.
● First presentation to her PCP 9 months after initiation of herbal treatment	Jaundice was objectively confirmed
● Discontinuation of herbal treatment 9 months after treatment initiation and 3 months following emerging pruritus as the first symptom	Hospital admission the other day and discontinuation of the herbal treatment as HILI was suspected. Clinical evaluation with exclusion of alternative diagnoses including HEV. Causality assessment by RUCAM that provided a probable causality. Following cessation of herbal treatment, clinical symptoms and LTs rapidly improved to complete recovery. This case is best described as idiosyncratic HILI of the metabolic type.

Additional details are presented in a previous report [41]. Abbreviations: HEV, Hepatitis E Virus; HILI, Herb induced liver injury; PCP, Primary care provider; RUCAM, Roussel Uclaf Causality Assessment Method; LT, liver tests.

**Table 5:** Clinical characteristics of kava hepatotoxicity.

Items	Clinical Characteristics of Kava Hepatotoxicity
● Positive reexposure result	1. The existence of kava hepatotoxicity has been verified by a positive reexposure test;
● RUCAM	2. RUCAM based causality for kava ± comedication was graded probable in 4 patients;
	3. In nine patients and thus in the majority of case, causality for kava ± comedication was possible and hence weak;
● Kava products	4. Kava hepatotoxicity may be caused by traditional aqueous kava extracts, commercial ethanolic and acetonic kava extracts, and kava-herbs mixtures;
● Risk factors	5. Daily overdose of kavalactones and prolonged treatment were common phenomena in patients with kava hepatotoxicity and considered as risk factors, although it occurred also with normal doses under recommended therapy duration of 3 months or less;
	6. Synthetic or herbal drugs and dietary supplements including herbal ones were comedicated with kava in the majority of cases and considered as risk factors;
	7. Additional risk factors included non-adherence to regulatory treatment recommendations, but not extraction media or solubilizers, used for the manufacturing of the kava drug;
● Age and Gender	8. The ages of the 14 patients with a probable causality for kava ± comedication or a highly probable causality for kava ranged from 14 to 60 years, and the ratio of females: males was 6:1;
● Hepatocellular injury	9. High serum activities were found for ALT but not for ALP, suggesting a hepatocellular injury type in kava hepatotoxicity;
● Liver histology	10. Histology showed predominantly liver cell necrosis and hepatitis;
● Pathogenetic type of liver injury	11. Depending on the kava product used, kava hepatotoxicity represents primarily the idiosyncratic type of injury with the metabolic subtype but secondarily also the intrinsic type of injury; the intrinsic and thereby predictable type of hepatotoxicity applies to those patients who might have used one of the few extracts containing kava of inappropriate quality with toxic properties, or who took kava with daily overdose or prolonged;
● Incidence	12. Valid epidemiology data are lacking, and the incidence of kava hepatotoxicity cannot be accurately calculated, but appears to be low.

Data are derived from a previous report [80,81] and consider other analyses and publications [82,83,84,85,86,87,88,89,90,91,92]. Abbreviations: ALP, Alkaline phosphatase; ALT, Alanine aminotransferase; RUCAM, Roussel Uclaf Causality Assessment Method.

**Table 6:** Clinical characteristics of the hepatic sinusoidal obstructive syndrome (HSOS) caused by *Gynura segetum* containing unsaturated PAs.

Conditions	Results
● Cohort	*n* = 116
● Gender	Males 57 Females 56 (NA 3)
● Age	17–76 years
● Ascites	115/116 cases
● Hepatomegaly	104/113 cases
● Jaundice	95/113 cases
● ALT elevation	47/60 cases (NA 56 cases)
● AST elevation	50/58 cases (NA 58 cases)
● Outcome	Recovery 75 cases Chronicity 27 cases Death 11 cases (NA 3 cases)

Data from Gao *et al.*, 2012 [28]. Abbreviations: ALT, Alanine aminotransferase; AST, Aspartate aminotransferase; NA, Not available; PAs, Pyrrolizidine alkaloids.

**Table 7:** Core elements and advantages of RUCAM.

Items	RUCAM
● Time frame of latency period (score)	+
● Time frame of dechallenge (score)	+
● Recurrent ALT or ALP increase (score)	+
● Definition of risk factors (score)	+
● All comedications (score)	+
● Individual comedication (score)	+
● Search for individual alternative causes (score)	+
● Verified exclusion of specific alternative causes (score)	+
● All specifically assessed HAV, HBV, HCV, HEV (score)	+
● All specifically assessed CMV, EBV, HSV, VZV (score)	+
● Evaluation of cardiac hepatopathy (score)	+
● Liver and biliary tract imaging (score)	+
● Color Doppler sonography of liver vessels (score)	+
● Prior known hepatotoxicity (score)	+
● Search for unintended reexposure (score)	+
● Definition of unintended reexposure (score)	+
● Qualified criteria of unintended reexposure (score)	+
● Laboratory hepatotoxicity criteria	+
● Laboratory hepatotoxicity pattern	+
● Hepatotoxicity specific method	+
● Structured, liver related method	+
● Quantitative, liver related method	+
● Validated method (gold standard)	+
● Worlwide use	+
● Use by international registries	+
● Use by regulatory agencies	+
● Use by HILI case reports	+
● Use by HILI case series	+
● Transparent documentation	+

Data are adapted from a previous report [12]. Abbreviations: ALT, Alanine aminotransferase; ALP, Alkaline phosphatase; CMV, Cytomegalovirus; EBV, Epstein Barr Virus; HAV, Hepatitis A Virus; HBV, Hepatitis B Virus; HCV, Hepatitis C Virus; HEV, Hepatitis E Virus; HILI, Herb induced liver injury; HSV, Herpes simplex Virus; RUCAM, Roussel Uclaf Causality Assessment Method; VZV, Varicella zoster Virus.

**Table 8:** Conditions of unintentional reexposure tests in HILI cases.

Reexposure Test Result	Hepatocellular Injury		Cholestatic or Mixed Liver Injury	
	ALTb	ALTr	ALPb	ALPr
● Positive	<5N	≥2ALTb	<2N	≥2ALPb
● Negative	<5N	<2ALTb	<2N	<2ALPb
● Negative	≥5N	≥2ALTb	≥2N	≥2ALPb
● Negative	≥5N	<2ALTb	≥2N	<2ALPb
● Uninterpretable	<5N	n.a.	<2N	n.a.
● Uninterpretable	n.a.	≥2ALTb	n.a.	≥2ALTb
● Uninterpretable	n.a.	n.a.	n.a.	n.a.

Conditions and criteria for an unintentional reexposure test are described in a previous report [12]. Accordingly, required data for the hepatocellular type of liver injury are the ALT levels just before reexposure, referred to as baseline ALT or ALTb, and the ALT levels during reexposure, referred to as ALTr. Response to reexposure is positive, if both criteria are met: first, ALTb is below 5N with N as the upper limit of the normal value, and second, after reexposure, ALT should increase to at least twice the baseline ALT value (ALTr ≥ 2ALTb). Other variations are evaluated as negative or uninterpretable results. For the cholestatic (±hepatocellular) type of liver injury, corresponding values of ALP are to be used instead of ALT. Abbreviations: ALP, Alkaline phosphatase; ALT, Alanine aminotransferase; HILI, Herb induced liver injury; N, Upper limit of Normal; n.a., not available.

**Table 9:** Causality assessment by RUCAM and/or positive reexposure tests in cases with assumed herbal hepatotoxicity by Traditional Chinese Medicine (TCM).

Herbal TCM	RUCAM Based Causality	Reexposure Based Causality
● Bai Xian Pi	+	−
● Bo He	+	−
● Ci Wu Jia	+	−
● Chuan Lian Zi	+	−
● Da Huang	+	−
● Gan Cao	+	−
● Ge Gen	+	−
● Ho Shou Wu	+	−
● Huang Qin	−	−
● Hwang Geun Cho	−	+
● Ji Gu Cao	−	+
● Ji Xue Cao	−	−
● Jin Bu Huan	+	+
● Jue Ming Zi	+	−
● Jiguja	+	−
● Kudzu	−	−
● Ling Yang Qing Fei Keli	+	−
● Lu Cha	+	+
● Ma Huang	−	+
● *Polygonum multiflorum*	+	+
● Rhen Shen	+	−
● Shou Wu Pian	+	+
● Shan Chi	+	−
● Shen Min	+	−
● Syo Saiko To	+	+
● Xiao Chai Hu Tang	−	+
● Zexie	+	−
● Zhen Chu Cao	+	−

This cohort study consisted of patients with suspected herbal hepatotoxicity by Traditional Chinese Medicine (TCM), using data of published reports for causality assessment by RUCAM, positive reexposure tests meeting specific criteria, or both. Respective details and references are provided in recent reports [23,100,128,129]. Abbreviations: RUCAM: Roussel Uclaf Causality Assessment Method.

**Table 10:** Confounding variables as examples in case reports of HILI.

Problematic Items as Confounding Variables for Causality Assessment in some Cases	References for few HILI Cases as Examples
● Problematic plant authentication	[14,16,22,25,26,27,28,29,105,106,107]
● Unspecified plant part	[107]
● Lacking herbal product identification	[106,107]
● Unidentified indication of herbal treatment	[107]
● Unassessed daily dosage	[107]
● Unidentified date of product start	[107]
● Unidentified date of product stop	[107]
● Unclear time to onset	[106,107]
● Unconfirmed herbal product purchase	[49,50]
● Unconfirmed herbal product use	[49,50]
● Unassessed comedication by drugs	[48,49,50]
● Use of many herbal dietary supplements	[49,50,107]
● Unreported initial ALT value	[107]
● Unreported initial ALP value	[107,135]
● Unconsidered HAV	[50,107,135]
● Unconsidered HBV	[50,99,106,107,135]
● Unconsidered HCV	[50,99,106,107,135]
● Unconsidered HEV	[48,49,50,99,106,107]
● Unconsidered CMV	[50,99,106,107]
● Unconsidered EBV	[48,49,50,99,106,107]
● Unconsidered HSV	[48,49,50,99,106,107]
● Unconsidered VZV	[50,99,106,107]
● Incomplete imaging data	[99,106,107]
● Ignored hepatotoxicity criteria	[107]
● Causality assessment by non-RUCAM	[24,94,99,105,107,112,120]

Abbreviations: ALT, Alanine aminotransferase; ALP, Alkaline phosphatase; CMV, Cytomegalovirus; EBV, Epstein Barr Virus; HAV, Hepatitis A Virus; HBV, Hepatitis B Virus; HCV, Hepatitis C Virus; HEV, Hepatitis E Virus; HILI, Herb induced liver injury; HSV, Herpes simplex Virus; RUCAM, Roussel Uclaf Causality Assessment Method; VZV, Varicella zoster Virus.

**Table 11:** Listing compilation of TCM herbs and herbal products with reported hepatotoxicity.

Chinese Name	Botanical Names, Ingredients
● Ai Ye	*Artemisia argyi*
● An Shu Ling	*Lycopodium serratum* or rarely, *Corydalis* specie*s*, *Panax ginseng*, Pseudo ginseng, or two species of *Stephania*
● Bai Fang	*Angelica sinensis*, *Cyperus rotundus*, Ginseng, *Ligusticum wallichii*, *Paeonia alba*, *Rehmannia glutinosa*
● Bai Shi Wan	*Atractylis*, *Carthamus tinctorius*, *Dalbergia odorifera*, *Dioscorea bulbifera*, *Glycyrrhiza*, *Lithospermum erythrorhizon*, *Paeonia suffruticosa*, *Polygonum multiflorum*, *Psoralea corylifolia*, *Salvia miltiorrhiza*; *Endoconcha sepiae*, *Ganoderma lucidum* (mushroom)
● Bi Ma Zi	*Rhicinus communis*, *Chaenomeles*, *Codonopsis pilosula*, *Notopterygium*, *Polygonum multiflorum*, *Rehmannia*, *Schisandra*
● Bo He	*Mentha haplocalyx*
● Bo Ye Qing Niu Dan	*Tinospora crispa*
●Bofu Tsu Sho San	*Angelica*, *Atractylis*, *Cnidium*, *Gardenia*, *Ephedra*, *Forsythia*, *Glycyrrhhiza*, *Gypsum fibrosum*, *Ledebouriella*, *Mentha*, *Paeonia*, *Platycodon*, *Rheum*, *Schizonepeta*, *Scutellaria*, *Zingiber*; Kadinum (talcum powder), sodium sulfuricum
● Boh Gol Zhee	*Psoralea corylifolia*
● Cang Er Zi	*Xanthium sibiricum*
● Chang Shan	*Dichora febrifuga Lour*
● Chai Hu	*Bupleurum falcatum*
● Chaso	*Camellia sinensis*, *Cassia tora* (syn. *Senna*), *Crataegus*, *N*-nitroso-fenfluramine
● Chi R Yun	*Breynia officinalis*
● Chinese herbal mixtures (various)	*Dictamnus dasycarpus*, *Gentiana scabra*, *Hedyotis diffusa*, *Paeonia suffructicosa*, *Paris polyphylla*, *Rehmannia glutinosa*, S*milax glabra*, *Sophora subprostrata*; *Angelica sinensis*, *Bupleurum chinese*, *Dictamnus dasycarpus*, *Paeonia suffructiosa*, *Philodendron chinese*, *Saposhnikovia divaricata*,*Shisandra chinesis*, *Shizonepeta tenuifolia*, *Tribulus terrestris*; *Cocculus trilobus*, *Dictamnus dasycarpus*, *Eurysolen gracilis*, *Glycyrrhiza*, *Lophatherum*, *Paeonia*, *Potentilla*, *Rehmannia glutinosa*; *Alisma plantago aquatica*, *Artemisia capillaris*, *Bupleurum*, C*hrysanthemum morifolium*, *Circuma*, *Gardenia jasminoidis*, *Gentiana scabra*, *Glycyrrhiza*, *Magnolia*, *Paeonia*, *Plantago asiatica*, *Saussurea lappa*
● Chuan Lian Zi	*Melia toosendan*
● Ci Wu Jia	*Acanthopanax senticosus*
● Da Chai Hu Tang	*Bupleurum falcatum*, *Ginseng*, *Glycyrrhiza glabra*, *Pinellia*, *Scutellaria*, *Zingiber officinale*, *Zizyphus jujuba*
● Da Huang	*Rheum palmatum*
● Du Huo	*Angelica archangelica*
● Fu Fang Qing Dai Wan	Angelica dahurica, *Isatis indigotica* (Indigo naturalis), *Massa medicata fermentata* (yeast), *Salvia milthiorrhiza*, *Smilax glabra*
● Gan Cao	*Glycyrrhiza uralensis*, syn. Liquorice
● Ge Gen	*Pueraria lobata*, syn. Arrowroot
● He Huan Pi	*Albizia julibrissin*
● Ho Shou Wu	*Polygonum multiflorum*, syn. He Shou Wu
● Hu Bohe You	*Mentha pulegium*, syn. Pennyroyal oil
● Hu Zhang	*Polygonum cuspidatum*
● Huang Qin	*Scutellaria baicalensis*
● Huang Yao Zi	*Dioscorea bulbifera*
● Hwang Geun Cho	*Corydalis speciosa*
● Ji Gu Cao	*Abrus cantoniensis*
● Ji Ji	*Chloranthus serratus*
● Ji Xue Cao	*Centella asiatica*, syn. Gotu Kola
● Jiguja	*Hovenia dulcis*
● Jin Bu Huan	*Lycopodium serratum* or rarely, *Corydalis species*, *Panax ginseng*, Pseudo ginseng, or two species of *Stephania*
● Jue Ming Zi	*Cassia obtusifolia*, syn. *Senna*
● Kamishoyosan	*Angelica sinensis*, *Atractylodes racea*, *Bupleurum falcatum*, *Gardenia*, *Glycyrrhiza glabra*, *Mentha haplocalyx*, *Moutan*, *Paeonia alba*, *Sclerotium Poriae Cocos*, *Zingiber officinale*
● Kudzu	*Pueraria thunbergiana*
● Ku Lian Zi	*Melia azedarach*
● Lei Gong Teng	*Tripterygium wilfordii* Hook
● Long Dan Xie Gan Tang	*Acebia*, *Alisma*, *Angelica sinensis*, *Bupleurum*, *Gardenia*, *Gentiana*, *Glycyrrhiza*, *Plantago*, *Rehmannia*, *Scutellaria*
● Lu Cha	*Camellia sinensis*, syn. Chinese green tea
● Ma Huang	*Ephedra sinica*
● Mao Guo Tian Jie Cai	*Heliotropium lasiocarpum*
● Onshido	*Aloe*, *Camellia sinensis*, *Crataegus*, G*ynostemma pentaphyllum makino*, *Raphanus*; *N*-nitroso-fenfluramine
● Qian Li Guang	*Senecio scandens*
● Ren Shen	*Panax ginseng*
● Sairei To	*Alisma*, *Atractylis*, *Bupleurum*, *Cinnamomum*, *Ginseng*, *Glycyrrhiza*, *Pinellia*, *Polyporus*, *Poria*, *Scutellaria*, *Zingiber*, *Zizyphus*
● Shan Chi	*Gynura segetum*
● Shang Lu	*Phytolacca acinosa*
● Shen Min	Black cohosh, Burdock, Cayenne pepper, *Ginkgo biloba*, Horse chestnut, *Piper nigrum*, *Polygonum multiflorum*, *uva ursi*; biotin, collagen (hydrolyzed), niacin, pantothenic acid, silica (from plant sources), soy isoflavones, vitamin A, vitamin B_6_
*●* Shi Can	*Teucrium chamaedrys*, syn. Germander
● Shi Liu Pi	*Pericarpium granati*
● ShouWu Pian	*Achyranthes bidentata*, *Cuscuta chinensis*, *Eclipta prostrata*, *Ligustrum lucidum*, *Lonicera japonica*, *Morus alba*, *Polygonum multiflorum*, *Psoralea corylifolia*, *Rehmannia glutinosa*, *Rosa aevigat*, *Sesemum indicum*, *Siegesbeckia orientalis*
● Tian Hua Fen	*Trichosanthes kirilowii*
● White flood	Qian Ceng Ta (*Huperzia serrata*), Wu Zhu Yu E*vodia rutaecarpa*); beet root, caffein, cocoa bean, vinpocetine (from *Vinca* plant); acesulfame potassium, calcium silicate, carnitine tartrate, Carno-Syn^®^ beta-alanine, citrulline, cryptoxanthin, folic acid, gamma-aminobutyric acid (GABA), glucuronolactone, selenium, L-norvaline, L-tyrosine, lutein, malic acid, ornithine, potassium gluconate, sucralose, sugar cane, watermelon flavor, zeaxanthin
● Wu Bei Zi	*Galla chinensis*
● Xi Shu	*Camptotheca acuminata*
● Xian Si Zi	*Abrus Precatorius*
● Xiao Chai Hu Tang	*Bupleurum falcatum*, *Ginseng*, *Glycyrrhiza glabra*, *Pinellia tuber*, *Scutellaria baicalensis*, *Zingiber officinale*, *Zizyphus jujuba*
● Yin Chen Hao	*Artemisia capillaris*
● Zexie	*Alisma orientalis*
● Zhen Chu Cao	*Phyllanthus urinaria*

Data are compiled from numerous reports [22,23,24,40,99,100,136,137,138,139,140,141,142,143,144,145,146,147,148,149,150,151,152,153,154,155,156,157,158,159,160,161,162,163,164,165,166,167,168,169,170,171,172,173,174,175,176,177,178,179,180,181,182,183,184,185,186,187,188,189,190,191,192,193,194,195,196,197,198,199,200,201], with individual references published previously [8,128]. In some cases, causality for individual herbs and herbal mixtures was established by using RUCAM. For other cases, information was fragmentary and did not necessarily allow a firm causal attribution.

**Table 12:** Listing compilation of herbs and herbal products with reported hepatotoxicity.

Search Terms	Botanical Names, Ingredients
● *Acacia catechu*	see Ayurvedic herbs
● Aloe	*Aloe perfoliata var. vera*
● *Amorphophallus Konjac*	see Hydroxycut^®^
● Arrowroot	*Maranta aruninacea* or *Tacca leontopetaloides*
● *Atractylis gummifera*	see Distaff thistle
● Ayurvedic herbs	*Psoralea corylifolia*, *Acacia catechu*, *Eclipta alba* or *Bacopa monnieri*, *Vetivexia zizaniodis*
● Babchi	*Psoralea corylifolia*, see also Ayurvedic herbs
● *Bacopa monnieri*	see Ayurvedic herbs
● *Boronia Sm.*	see Pro-Lean^®^
● Buchu tea	*Agathosma betulina*, *Agathosma crenulata*
● Bush tea	*Crotalaria species*
● *Callilepis laureola*	see Impila
● *Camellia sinensis*	see green tea, Exolise^®^, Hydroxycut^®^, X-elles^®^
● Cascara sagrada	*Rhamnus purshianus*
● *Cassia angustifolia*	see Senna
● *Centella asiatica*	see Gotu Kola, Pro-Lean^®^
● *Chamaerops humilis*	see Saw Palmetto
●Chaparral syn. Creosot	*Larrea tridentata*, *Larrea divariatica*
● *Chelidonium majus*	see Greater Celandine, Lycopodium similiaplex^®^
● *Chlorophora species*	see Kambala tea
● *Chrysanthemum leucanthemum*	see Oxeye Daisy
● *Citrus aurantium*	see X-elles^®^
● *Citrus paradisum*	see X-elles^®^
● *Cyrana scolymus*	see X-elles^®^
● *Cola nitida*	see Pro-Lean^®^
● Coltsfoot	*Tussilago farfara*
● Comfrey	*Symphytum officinale*, *Symphytum asperum*, *Symphytum uplandicum*
● *Compositae species*	see Indian herbs
● Creosot	see Chaparral
● *Crotalaria species*	see Bush tea, Rattlebox
● *Cyperus*	see Pro-Lean^®^
● Distaff thistle	*Atractylis gummifera*
● *Eclipta alba*	see Ayurvedic herbs
● *Emblica officinalis*	see Isabgol
● *Ephedra species*	*Ephedra californica*, *Ephedra sinica*
● Exolise^®^	*Garcinia cambogia*, *Gymnema sylvestre*, White kidney bean, *Camellia sinensis*, l-Carnitine fumarate, Calcium, Magnesium chelate, Chromium chelate, Conjugated linoleic acid, Chitosan
● *Fallopia multiflora*	see Pro-Lean^®^
● *Foeniculum amare*	see Herbalife^®^
● *Fucus vesiculosus*	see Pro-Lean^®^
● *Garcinia cambogia*	see Exilis^®^, Herbalife^®^, Hydroxycut^®^
● Germander	*Teucrium chamaedrys*, *Teucrium polium*
● *Ginkgo biloba*	seePro-Lean^®^
● *Ginseng*	see Bai Fang, Dai Saiko To, Pro-Lean^®^, Xiao Chai Hu Tang
● *Glycyrrhiza glabra*	See Dai Saiko To, Xiao Chai Hu Tang
● Gotu Kola	*Centella asiatica*
● Greater Celandine	*Chelidonium majus*, see also Lycopodium similiaplex^®^
● Green tea	*Camellia sinensis*, see also Lu Cha
● Groundsel syn. Senecio	*Senecio longilobus*, *Senecio species*
● Guaraná	*Paullinia cupana*
● *Gymnema sylvestre*	see Exilis ^®^, Hydroxycut^®^
● Hawthorn	see *Crataegus*
*● Hedeoma pulegoides*	see Pennyroyal
● Heliotropium	*Heliotropium eichwaldii*, *Heliotropium species*
● Herbalife^®^	*Solidaginis gigantea*, *Ilex paraguariensis*, *Petroselinum crispum*, *Garcinia cambogia*, *Spiraea*, *Matricaria chamomilla*, *Liquiritia*, *Foeniculum amare*, *Humulus lupulus*, Chromium, and various other ingredients
● Horse chestnut	see Venencapsan^®^, Venoplant^®^
● *Humulus lupulus*	see Herbalife^®^
● Hydroxycut^®^	*Camellia sinensis*, *Gymnema sylvestre*, *Amorphophallus Konjac*, *Paullinia cupana*, *Garcinia cambogia*, Caffeine, α-Lipoic acid, l-Carnitine, Calcium, Potassium, Chromium
● *Ilex paraguariensis*	see Herbalife^®^, Maté
● Impila	*Callilepis laureola*
● Indian herbs	*Compositae species*
● Iroko	see Kambala Tea
● Isabgol	*Plantago ovata*, *Emblica officinalis*
● Kambala Tea syn. Iroko	*Chlorophora excelsa*, *Chlorophora regia*
● Kava	*Piper methysticum*
● *Larrea divariatica*	see Chaparral
● *Larrea tridentata*	see Chaparral
● *Leucanthemum vulgare*	see Oxeye Daisy
● *Liquiritia*	see Herbalife^®^
● *Lycopodium serratum*	see Lycopodium similiaplex^®^, Wolf’s foot clubmass
● Lycopodium similiaplex^®^	*Lycopodium serratum*, *Chelidonium majus*
● *Maranta aruninacea*	see Arrowroot
● Maté	*Ilex paraguariensis*
● *Mentha pulegium*	see Pennyroyal
● Mistletoe	*Viscum album*
● *Monascus purpureus*	see Red Yeast Rice
● *Morinda citrifolium*	see Noni
● *Nerium oleander*	see Oleander
● Noni	*Morinda citrifolium*
● Oleander	*Nerium oleander*
● Oxeye Daisy	*Leucanthemum vulgare*, *Chrysanthemum leucanthemum*
● *Paullinia cupana*	see Guaraná, Hydroxycut^®^, Pro-Lean^®^
● Pennyroyal	*Mentha pulegium*, *Hedeoma pulegoides*
● *Petroselinum crispum*	see Herbalife^®^
● *Petroselinum sativum*	see X-elles^®^
● *Piper methysticum*	see Kava
● *Phaseolus vulgaris*	see Exilis^®^
● *Plantago ovata*	see Isabgol
● Pro-Lean^®^	Ma Huang, *Paullinia cupana*, *Cola nitida*, *Centella asiatica*, *Salix alba*, *Ginkgo biloba*, *Fucus vesiculosus*, *Boronia Sm.*, *Ginseng*, *Fallopia multiflora*, *Cyperus*, Bee pollen, Caffeine, l-Tyrosine, Chromium, Vanadium, Magnesium salicylat, Folic acid, Vitamin B_12_, and various other ingredients
● *Psoralea corylifolia*	see Ayurvedic herbs
● Pyrrolizidine alkaloid containing herbs	see Bush tea, see Comfrey, see Groundsel, see *Heliotropium species*, see Indian herbs, see Maté, see Rattlebox
● Rattlebox syn. C*rotalaria*	*Crotalaria species*
● Red Yeast Rice	*Monascus purpureus*
● *Rhamnus purshianu*	see Cascara sagrada
● Rooibos tea	*Aspalathus linearis*
● *Salix alba*	see Pro-Lean®
● Sassafra	*Sassafras albidum*
● Saw Palmetto	*Serenoa serpens*, *Chamaerops humilis*
● Skullcap	*Scutellaria* *lateriflora*, *Scutellaria* *species*
● *Scutellaria species*	see Skullcap
● *Senecio*	see Groundsel
● Senna	*Cassia angustifolia*
● *Serenoa serpens*	see Saw Palmetto
● *Solidaginis gigantea*	see Herbalife^®^
● *Spiraea*	see Herbalife^®^
● *Stonecrop*	*Sedum aizoon*
● *Symphytum*	see Comfrey
● *Tacca leontopetaloides*	see Arrowroot
● *Teucrium*	see Germander
● *Tussilago farfara*	see Coltsfoot
● Valerian	*Valeriana officinalis*
● *Valeriana officinalis*	see Valerian
● Venencapsan^®^	*Aesculus hippocastanum*, *Chelidonium majus*, *Melilotus officinalis*, Milfoil, *Silybum* Adans., *Taraxacum officinale*
● Venoplant^®^	*Aesculus hippocastanum*
● *Vetivexia zizaniodis*	see Ayurvedic herbs
● *Viscum album*	see Mistletoe
● Wolf’s foot clubmass	*Lycopodium serratum*
● X-elles^®^	*Petroselinum sativum*, *Citrus aurantium*, *Citrus paradisum*, *Cyrana scolymus*, *Camellia sinensis*

Data are collected from published reports [22,23,24,32,40,41,72,100,111,136,137,149,158,171,173,174,175,176,177,178,180,189,202,203,204,205,206,207,208,209,210,211,212,213,214,215,216,217,218,219,220,221,222,223,224,225,226,227,228,229,230,231,232,233,234,235,236,237,238,239,240,241,242,243,244,245,246,247,248,249,250,251,252,253,254,255,256,257,258,259,260,261,262,263,264,265,266,267,268,269,270,271,272,273,274,275,276,277,278,279,280,281,282,283,284,285,286,287,288,289,290,291,292,293,294,295,296,297,298,299,300,301,302,303,304,305,306,307,308,309,310,311], and specific references for each herb are found in a recent report [8]. For few of the herbs, causality was ascertained by using RUCAM or positive test results of unintentional reexposure. For most herbs, causality was not firmly established and is open for discussion.
